# Efficacy of acupuncture versus sham acupuncture for postprandial distress syndrome: study protocol for a randomized controlled trial

**DOI:** 10.1186/s13063-018-3051-3

**Published:** 2019-01-18

**Authors:** Ya-Quan Hou, Xin Zhang, Jian-Feng Tu, Yang Zheng, Jing-Wen Yang, Mirim Kim, Hui Hu, Li-Qiong Wang, Jing-Jie Zhao, Wei Zhou, Jun Wang, Xuan Zou, Yu Wang, Guang-Xia Shi, Cun-Zhi Liu

**Affiliations:** 1 Department of Acupuncture and Moxibustion, Dongfang Hospital, Beijing University of Chinese Medicine, Fengtai District, Beijing, China; 2 Department of Acupuncture and Moxibustion, Beijing Hospital of Traditional Chinese Medicine Affiliated to Capital Medical University, Dongcheng District, Beijing, China; 3 Department of Traditional Chinese Medicine, Beijing Friendship Hospital, Capital Medical University, Xicheng District, Beijing, China; 4 Department of Traditional Chinese Medicine, Huguosi Hospital of Chinese Medicine, Beijing University of Chinese Medicine, Xicheng District, Beijing, China; 5 Department of Acupuncture and Moxibustion, Dongzhimen Hospital, Beijing University of Chinese Medicine, Dongcheng District, Beijing, China

**Keywords:** Acupuncture, Functional dyspepsia, Postprandial distress syndrome, Randomized controlled trial, Sham acupuncture

## Abstract

**Background:**

Postprandial distress syndrome (PDS) has a considerable impact on quality of life. Our previous pilot trial suggested that acupuncture might be a potential treatment option for PDS. We will conduct this large trial to determine the efficacy of acupuncture versus sham acupuncture for PDS.

**Methods/design:**

A total of 280 eligible patients who meet the Rome IV criteria for PDS will be randomly allocated to either the acupuncture group or the sham acupuncture group. Each patient will receive 12 sessions over four weeks. The primary outcomes will be the response rate of overall treatment effect (OTE) and the elimination rate of all three cardinal symptoms (postprandial fullness, upper abdominal bloating, and early satiation) at four weeks after randomization. Secondary outcomes will include assessments of the severity of dyspepsia symptoms and disease-specific quality of life at weeks 4, 8, and 16 after randomization. All patients who receive randomization will be included in the intent-to-treat analysis.

**Discussion:**

The finding of this trial will provide high-quality evidence on the efficacy of acupuncture for treatment of PDS. Results of this research will be published in peer-reviewed journals.

**Trial registration:**

ISRCTN Registry, ISRCTN12511434. Registered on 31 March 2017.

**Electronic supplementary material:**

The online version of this article (10.1186/s13063-018-3051-3) contains supplementary material, which is available to authorized users.

## Background

Functional dyspepsia (FD) is a common functional gastrointestinal disorder [1]. In America, epidemiological surveys suggest that 12–15% of people have symptoms suggestive of FD [2]. About one-quarter of these people seek medical assistance [3]. Although FD is not a life-threatening disease [4, 5], quality of life (QoL) is considerably affected [6, 7]. The high rate of absenteeism and low productivity at work have substantial implications for patients, healthcare organizations, and society [8–10].

Trials testing treatment response by FD subgroup are urgently needed [11, 12]. Based on the Rome IV consensus, FD is subdivided into postprandial distress syndrome (PDS), characterized by postprandial fullness and early satiation, and epigastric pain syndrome (EPS), characterized by epigastric pain and burning [1]. There are different pathogenic mechanisms underlying these FD subgroups. PDS is associated with motility dysfunction and impaired gastric accommodation, while EPS is associated with a high prevalence of hypersensitivity to gastric distension [13]. PDS has a higher prevalence than EPS among the Asian population. Of 563 patients diagnosed with FD in Japan, 89% had PDS and 33% had epigastric pain syndrome, with an overlap in 22% [14]. It has been suggested that PDS patients may respond to a therapy that alters gastric motility [15], such as prokinetic drugs. However, these prokinetic drugs have been implicated in potential side effects, including parkinsonism syndrome and cardiac arrhythmia [16–18].

Acupuncture is widely accepted as an effective treatment option for gastrointestinal disorders in clinical practice [19]. A number of studies have shown that acupuncture is able to facilitate gastrointestinal motility [20, 21]. However, a recent meta-analysis of acupuncture for FD indicates that the efficacy of acupuncture for FD has not been verified because of poor quality of the trials [22]. To our knowledge, rare studies have been designed for PDS with acupuncture especially. A retrospective trial of FD shows that patients with PDS respond better to the acupuncture therapies than those with EPS [23]. The main objective of this trial is to evaluate the efficacy of acupuncture versus sham acupuncture for PDS.

## Methods

### Study design

**Fig. 1:**
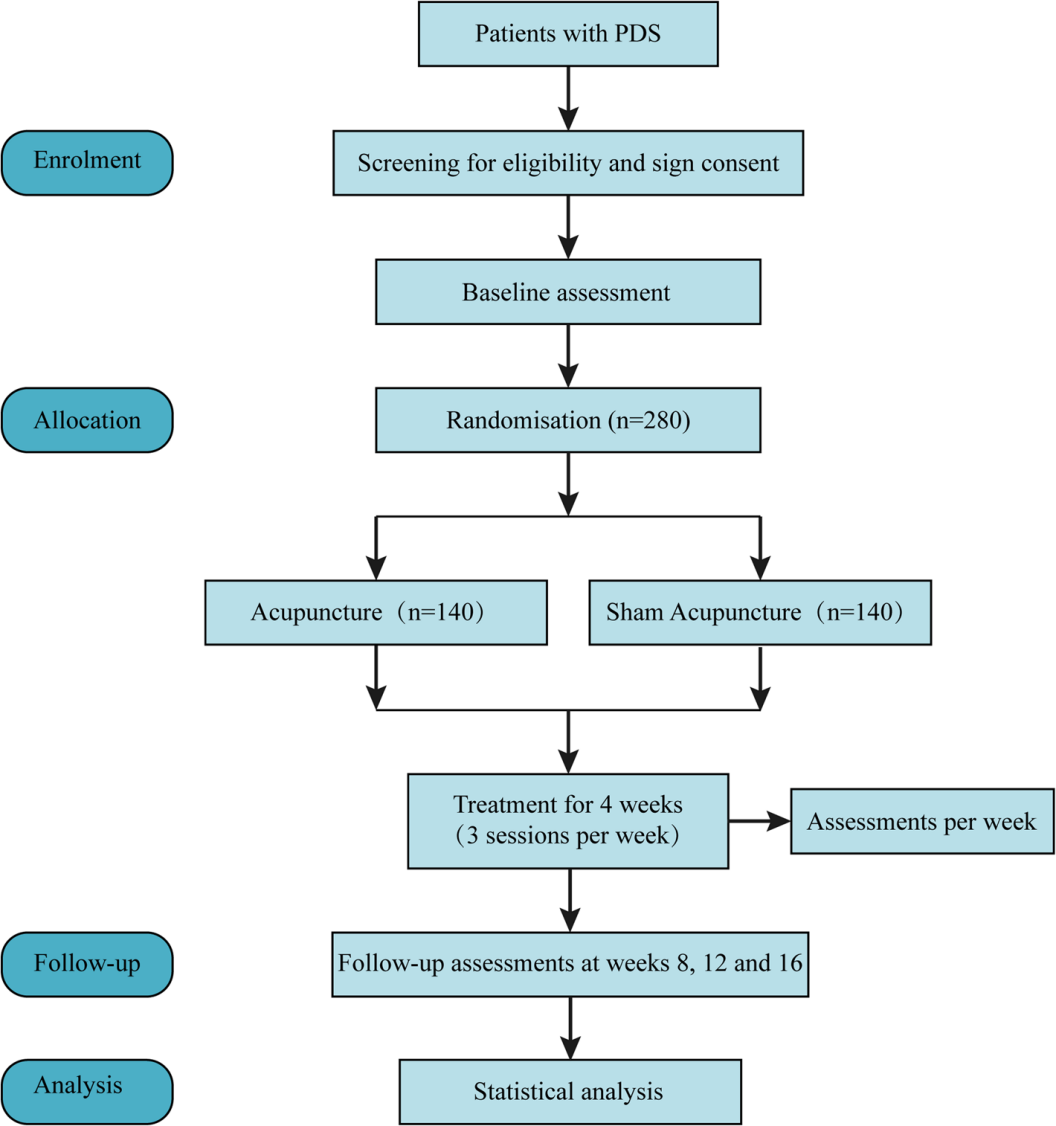
Flow diagram

This multi-center, randomized, participant-blind, sham-controlled trial will be conducted at five hospitals in China: Dongfang Hospital Affiliated to Beijing University of Chinese Medicine; Dongzhimen Hospital Affiliated to Beijing University of Chinese Medicine; Huguosi Hospital of Chinese Medicine Affiliated to Beijing University of Chinese Medicine; Beijing Hospital of Traditional Chinese Medicine Affiliated to Capital Medical University; and Beijing Friendship Hospital Affiliated to Capital Medical University. The study protocol has been approved by ethics committees at all participating hospitals, will follow the Declaration of Helsinki, and will be reported based on SPIRIT guidelines (Additional file 1) [24]. This trial has been registered with ISRCTN at Current Controlled Trials (ISRCTN12511434). A flow diagram of the trial is shown in Fig. 1.

### Patient recruitment

Patients meeting the Rome IV PDS criteria will be recruited primarily through advertisements on hospital social Internet media (WeChat) and newspaper, outpatient clinics, and publicity at community service centers. All patients will be required to provide written informed consent before randomization. Carbon-13 urea breath test will be routinely performed for patients due to the high prevalence of *H. pylori* infection in China.

#### Inclusion criteria

1. Aged 18–65 years (either sex);
2. One or more of the following symptoms: postprandial fullness; upper abdominal bloating; or early satiation;
3. Normal esophagogastroduodenoscopy results within one year;
4. No acupuncture treatment in the last one month;
5. No participation in any other research in the last two months.

#### Exclusion criteria

1. Dyspepsia symptoms caused by any serious or malignant disease, such as liver cirrhosis, heart failure, or gastrointestinal tumors;
2. Surgery related to the gastrointestinal tract;
3. Taking drugs which might affect dyspepsia, such as anti-secretary drugs, antacids, prokinetics, non-steroidal anti-inflammatory drugs, and antidepressant drugs within one month of participating in the trial;
4. Difficulties in attending the trial, such as serious mental and physiological illness, dementia, or illiteracy;
5. Severe coagulopathy;
6. Drug or alcohol abuse;
7. Pregnancy or breastfeeding.

### Randomization and allocation concealment

All eligible patients will be randomly assigned to the acupuncture group or sham acupuncture group in a 1:1 ratio with a central web-based randomization (Beijing Guide Technology Co., Ltd.). The blocked randomization sequence will be computer-generated with the SAS 9.3 software by a professional statistician, who is not involved in the implementation and statistical analysis of the trial. The clinical research coordinator will be responsible for enrolling patients, obtaining informed consent and requesting randomization.

### Masking

Due to the nature of acupuncture, masking of acupuncturists is quite difficult to achieve. Patients, outcome assessors, and statisticians who perform the statistical analyses will be blinded to group assignment. Participant’s allocated intervention will be not revealed until the statistical analysis is completed.

### Interventions

Treatment will be performed by licensed acupuncturists who have at least three years of experience in acupuncture. All the acupuncturists will be trained how to locate acupoints and non-acupoints, puncture, and manipulate needles before the trials. Sterile disposable acupuncture needles (length: 25–40 mm, diameter: 0.25 mm; Hwatuo, Suzhou, China) will be used. Both the acupuncture and sham acupuncture treatments will consist of 12 sessions with 20-min duration over four weeks (three sessions per week, ideally every other day). Acupuncture will be discontinued if the patients suffer from any adverse events (AEs). Other treatments which may affect the dyspepsia symptoms will be prohibited, such as antacids, prokinetics, non-steroidal anti-inflammatory drugs, antidepressant drugs, and so forth.

#### Acupuncture

**Fig. 2:**
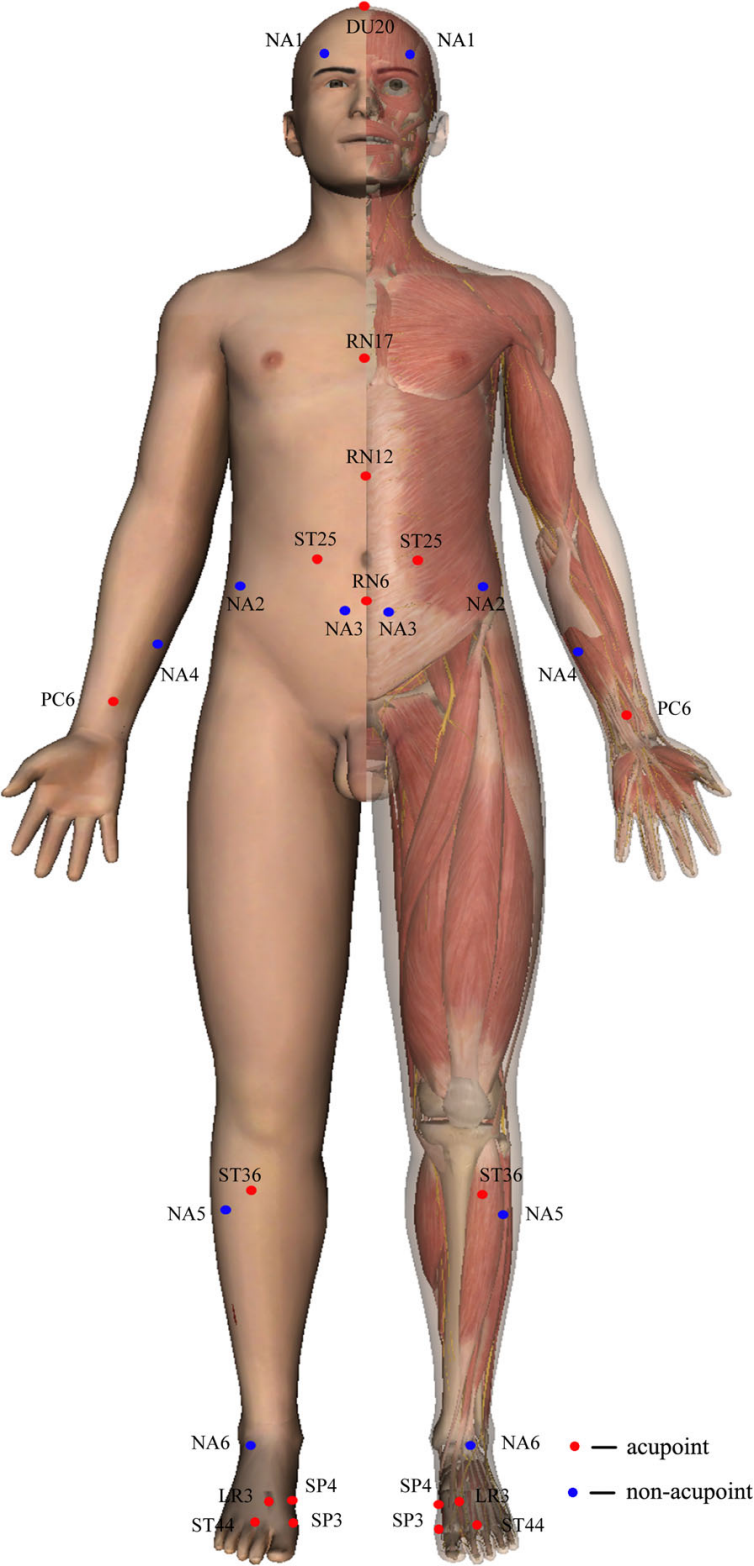
Locations of acupoints and non-acupoints

Patients allocated to the acupuncture group will take treatment with needles inserted at the prespecified acupuncture points. The protocol including obligatory and additional acupoints was developed from the clinical experience of acupuncture experts. The obligatory acupoints included *Baihui (DU20)*, *Danzhong (RN17)*, *Zhongwan (RN12)*, and *Qihai (RN6)*, and bilateral *Tianshu (ST25)*, *Neiguan (PC6)*, *Zusanli (ST36)*, and *Gongsun (SP4)*. According to different Traditional Chinese Medicine syndromes, additional acupoints could be chosen individually: *Taibai (SP3)* for weakness of the *qi* of the spleen and stomach; *Taichong (LR3)* for depression of the *qi* of the liver; and *Neiting (ST44)* for damp-heat in the stomach. All acupoints are localized according to the WHO Standard Acupuncture Locations and exhibited in Table 1 and Fig. 2. Manipulations of twirling, lifting, and thrusting will be performed on all needles for at least 30 s to reach *De qi* (a compositional sensation including soreness, numbness, distention, and heaviness), which is believed to be an essential component for acupuncture efficacy.

**Table 1:** Locations of acupoints in acupuncture group

Acupoints	Locations
Baihui (*DU20*)	On the midline of the head, 7 cun^a^ directly above the midpoint of the posterior hairline
Danzhong (*RN17*)	On the anterior midline, on the level of the 4th intercostal space, at the midpoint of the line joining the two nipples
Zhongwan (*RN12*)	On the anterior midline, 4 cun above the umbilicus
Tianshu (*ST25*)	On the same level of the umbilicus, and 2 cun lateral to the anterior midline
Qihai (*RN6*)	On the anterior midline, 1.5 cun below the umbilicus
Neiguan (*PC6*)	On the line joining Daling (*PC7*) and Quze (*PC3*), between the tendons of palmaris longus and flexor carpi radialis, 2 cun above the transverse crease of the wrist
Zusanli (*ST36*)	3 cun directly below Dubi (*ST35*), and one finger-breadth lateral to the anterior border of the tibia
Gongsun (*SP4*)	On the medial aspect of the foot, anteroinferior to the base of the first metatarsal bone, at the border between the red and white flesh
Taibai (*SP3*)	On the medial aspect of the foot, in the depression proximal to the first metatarsophalangeal joint, at the border between the red and white flesh
Taichong (*LR3*)	In the depression anterior to the junction of first and second metatarsal bones
Neiting (*ST44*)	On the dorsum of the foot, between the second and third toes, posterior to the web margin, at the border between the red and white flesh

^a^1 cun (≈20 mm) is defined as the width of the interphalangeal joint of patient’s thumb

#### Sham acupuncture

A superficial skin penetration (2–3 mm in depth) at non-acupoints will be performed in the sham acupuncture group, without needle manipulation for *De qi*. Non-acupoints are away from conventional acupoints or meridians and are shown in Table 2 and Fig. 2.

**Table 2:** Locations of non-acupoints in sham acupuncture group

Non-acupoints	Locations
Non-acupoint 1	In the middle of Touwei (*ST8*) and Yuyao (*EX-HN4*) points
Non-acupoint 2	2 cun^a^ above the anterior superior iliac spine
Non-acupoint 3	2 cun below the umbilicus, and 1.0 cun lateral to the anterior midline. cun below the umbilicus, and 1 cun lateral to the anterior midline
Non-acupoint 4	In the middle of the medial epicondyle of the humerus and the styloid process of ulna
Non-acupoint 5	3 cun below Yanglingquan (*GB34*), between the gallbladder and bladder meridian
Non-acupoint 6	In the middle of Qiuxu (*GB40*) and Jiexi (*ST41*) points

^a^1 cun (≈20 mm) is defined as the width of the interphalangeal joint of patient’s thumb

Although it is difficult to set an eligible placebo control, superficial insertion at non-acupoints is the most commonly used approach for administering sham treatments among acupuncture trials on the basis of a literature review [25]. And this study will exclude those received acupuncture treatment in the last one month. This population can most probably distinguish sham acupuncture from acupuncture. The same control, shallow needling at non-acupoints, was adopted and is successful to mask Chinese participants with chronic severe functional constipation [20]. Furthermore, all participants will be asked to guess which treatment they have received to test the patient-blinding effects.

### Outcomes

#### Primary outcomes

The two primary outcomes are the response rate based on overall treatment effect (OTE) and the elimination rate of all three cardinal symptoms: postprandial fullness; upper abdominal bloating; and early satiation (no symptoms) at the end of treatment (four weeks after randomization) [26].

When assessing OTE, outcome assessors will evaluate patients using a 7-point Likert scale. The question is “How were your gastric symptoms during the past week in comparison with the baseline period?”, which closely resembles the way physicians evaluate treatment benefit in clinical practice. The answers will include “extremely improved,” “improved,” “slightly improved,” “not changed,” “slightly aggravated,” “aggravated,” and “extremely aggravated” [26]. Patients who answer “extremely improved” or “improved” will be considered as responders. Although there is no universally accepted primary outcome established for therapeutic trials in FD, OTE is a frequently used outcome [27], which has been used as the primary outcome in several trials [28–30].

The elimination rate of three cardinal symptoms was added as another primary outcome. Because the U.S. Food and Drug Administration has argued against the use of OTE as a single primary outcome for its recall bias. After trial commencement, the Data and Safety Monitoring Board (DSMB) suggested that we could use combined primary outcomes for our study. Similarly, the pattern of two primary outcomes was adopted in a study evaluating acotiamide in FD [26]. The elimination rate is based on the severity of dyspepsia symptoms [31] and is defined as the proportion of patients whose scores of postprandial fullness, upper abdominal bloating, and early satiation all decline to 0 [26].

Acupuncture will be considered as an effective therapy, only if both primary outcomes achieve significant difference to avoid type I error accumulation. All protocol amendments are described in detail in Additional file 2.

#### Secondary outcomes

OTE at other time points

Response rate according to OTE and the elimination rate of all three cardinal symptoms will also be measured at weeks 8, 12, and 16 after randomization.

## Severity of dyspepsia symptoms

The severity of eight dyspepsia symptoms, including postprandial fullness, early satiation, upper abdominal bloating, epigastric pain, epigastric burning, nausea, vomit, and belching, will be assessed at baseline, once every week in treatment period and at weeks 8, 12, and 16. The severity of each symptom is scored as asymptomatic (0 point), mild (1 point), moderate (2 points), or severe (3 points) [31].

## Quality of life (QoL)

Disease-specific QoL will be assessed at baseline and at weeks 4, 8, and 16 after randomization using 25-item Nepean Dyspepsia Index (NDI) [32, 33], which consists of four domains: interference (13 items); know/control (7 items); eat/drink (3 items); and sleep/disturb (2 items). Each item is measured with a 5-point Likert scale ranging from “not at all” to “extremely.” Higher scores indicate a better QoL.

## Depression and anxiety

Anxiety is linked to PDS [34]. Depression and anxiety symptoms will be assessed at baseline and at weeks 4, 8, and 16 after randomization using the Hospital Anxiety Depression Scale (HADS) [32, 33]. HADS is a self-reported inventory including anxiety and depressive subscales, and each subscale consists of seven questions with scores in the range of 0–21. Higher scores indicate worse symptoms.

## Blinding assessment

To test the patient-blinding effects, all patients will be asked to guess whether they have received acupuncture or sham acupuncture within 5 min after the first treatment session and the sixth treatment session in week 2.

## Credibility and expectancy

Credibility and expectancy of patients will be assessed using the Credibility/Expectancy Questionnaire [35] within 5 min after the first treatment.

**Fig. 3:**
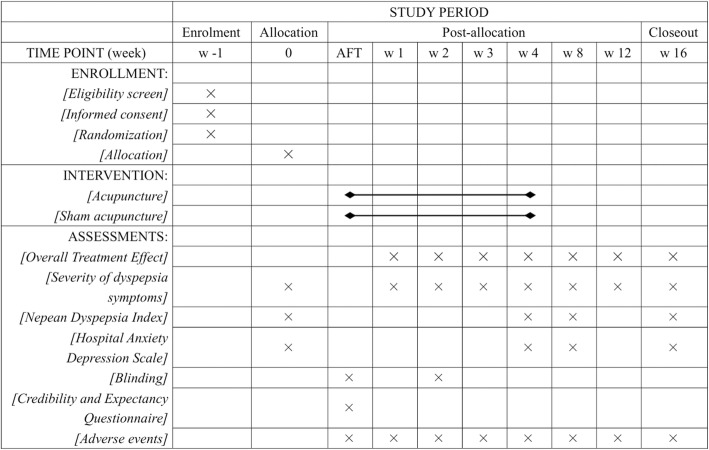
Schedule of enrollment, intervention, and assessments of this study protocol. AFT after the first treatment, w week

The schedule of enrolment, intervention, and assessments is shown in Fig. 3.

### Adverse events

Any AEs will be monitored and recorded by patients, acupuncturists, and outcome assessors using a preassigned questionnaire throughout the trial. Based on their potential association with the acupuncture needling procedure, AEs will be categorized by acupuncturists and related specialists as treatment-related or not within 24 h of occurrence. Common treatment-related AEs include local bleeding, subcutaneous hematoma, itching at the sites of needle insertion, continuous post-needling pain, dizziness, and so on.

### Data management

All researchers including acupuncturists, outcome assessors, and statisticians will receive training regarding data management. Data will be inputted into the electronic case report form (eCRF) which will be established before recruitment. The clinical research associates are responsible for verifying the accuracy of data. Online monitoring will be used in this trial. With the support of the “check” function in the eCRF, dynamic management will be implemented to ensure that the data are collected completely, promptly, and accurately. Data lockup will be implemented by the data management team on completion of the study. The researchers will then be unable to modify the data.

All research documents, including both the paper files and electronic documents, will be preserved for at least five years after publication. If reviewers or readers have any questions regarding our published data, they can contact the corresponding author for access to the original data. The private information of patients—including name, telephone number, and ID number—will be anonymous to ensure participant confidentiality.

In addition, an independent Data and Safety Monitoring Board (DSMB) will be established to review and interpret data generated from the study. The DSMB will review the progress of the trial every three months independently from investigators and the sponsor and decide on any premature closure of the study. The members of Data and Safety Monitoring Board are listed in Additional file 3.

### Quality control

The trial protocol is reviewed and revised by the experts in acupuncture, gastroenterology, methodology, and statistics. Prespecified standard operating procedure—including intervention, details in filling eCRF, assessment of outcomes, data management—will be used to train the related staff. An inspection plan will be designed for quality control. Health education and timely follow-up will also be made to improve the compliance of patients. For participants who discontinue acupuncture or deviate from protocol, the outcome assessors will still try to assess the outcomes.

### Statistical methods

#### Sample size

Since acupuncture is a complex intervention, it is different from drugs. The efficacy of acupuncture will be altered, if the acupoints in the study are changed. For this reason, literature data speculation is not adopted in this trial. Based on previous clinical experience, the proportions of acupuncture and sham acupuncture responders for OTE are expected to be 55% and 35%, respectively. For elimination of all the three symptoms, the proportions are expected to be 26% and 10%, respectively. A two-sided significance level is 0.05. A sample size of 224 (112 patients in each group) was estimated to have at least 80% power to detect significant differences. The power was calculated as the product of the power of each endpoint: 82.5% and 84.3%. To compensate for a 20% attrition rate, the sample size expands to 140 patients in each group.

#### Statistical analysis

The statistical analysis will be performed by an independent statistician who is not aware of the group allocation. SPSS 21.0 statistical software (IBM SPSS Statistics, New York, USA), will be used for data analysis. The level of significance will be established at α < 0.05 with a two-sided test. Continuous data will be represented as the mean ± standard deviation or median (range), whereas categorical data will be represented by percentage.

All efficacy analyses will be performed using the intent-to-treat (ITT) approach. For the ITT analysis, the population will consist of all patients who have been randomized. Missing data will be imputed using the multiple imputation method. Continuous variables will be compared using a Student’s t-test or Wilcoxon rank-sum test as appropriate. Categorical variables will be compared using Fisher’s exact test or Wilcoxon rank-sum test as appropriate. A preplanned subgroup analysis according to *H. pylori* status will be performed for the primary outcomes.

A sensitivity analysis will be conducted for the primary outcome using per-protocol (PP) population, including only those patients who complete at least 10 sessions and have no major protocol violations (taking other drugs during the trial, not completing the eCRF as required, etc.).

## Discussion

PDS impacts patients’ QoL and causes a financial burden for society. This large trial will evaluate the efficacy of acupuncture versus sham acupuncture in improving the symptoms of PDS.

This trial meets the methodological demand of adequate randomization and allocation concealment, blinding of patients, outcome assessors, and statisticians. A suitable control group is critical for a well-designed clinical trial. On the basis of literature review, superficial insertion at non-acupoints is the most commonly used approach for administering sham treatments among acupuncture trials [26]. Additionally, the amount of acupuncture is intensive, which is similar to clinical practice in China. Needles will be stimulated manually for at least 30 s at each acupoint and retained in place for 20 min. The treatment frequency is three sessions weekly.

The limitation of our trial is recall bias of pre-treatment symptom severity at the assessment of overall OTE. However, OTE is a reliable and frequently used outcome [27], which has been used as the primary outcomes in several trials [28–30]. Second, the acupuncturists are not blinded for the nature of intervention. Moreover, the use of a fixed block randomization may lead to block size exposure, which may not prevent selection bias from the prediction of treatment allocation in patients. At the end of this trial, we hope the results will provide more reliable evidence and clarify the value of acupuncture as a treatment for PDS.

### Trial status

This trial is currently recruiting patients.

## Additional files

Additional file 1: Completed Standard Protocol Items: Recommendation for Interventional Trials (SPIRIT) 2013 Checklist: items addressed in this clinical trial protocol. (DOC 120 kb)

Additional file 2: Functional Dyspepsia Trial Cumulative Protocol Amendments. (DOC 14 kb)

Additional file 3: Members of the Data and Safety Monitoring Board. (DOC 17 kb)

## Abbreviations

DSMB: Data and Safety Monitoring Board
eCRF: Electronic case report form
EPS: Epigastric pain syndrome
FD: Functional dyspepsia
HADS: Hospital Anxiety Depression Scale
ITT: Intent-to-treat
NDI: Nepean Dyspepsia Index
OTE: Overall treatment effect
PDS: Postprandial distress syndrome
PP: Per-protocol
QoL: Quality of life
