# Supplementary material for: Efficacy of acupuncture versus sham acupuncture for postprandial distress syndrome: study protocol for a randomized controlled trial
Source: Trials. 2019 Jan 18;20:65. doi: 10.1186/s13063-018-3051-3 (PMC6339308; doi:10.1186/s13063-018-3051-3)
Supplement: Supplementary file 2 — Functional Dyspepsia Trial Cumulative Protocol Amendments. (DOC 14 kb) [file 13063_2018_3051_MOESM2_ESM.doc]

**Functional Dyspepsia Trial Cumulative Protocol Amendments**

1. There were personnel modifications made throughout the study duration, as needed. The study PI, Dr. Liu CZ, was deployed to Dongfang Hospital from Beijing Hospital of Traditional Chinese Medicine in 2018. Thus, Dongfang Hospital became the major center and Beijing Hospital of Traditional Chinese Medicine became the secondary center.
2. Elimination rate of three meal-related symptoms was added as another primary efficacy end point. Because the US Food and Drug Administration (FDA) has argued against the use of OTE as the single primary outcome in drug trials, for the inherent recall, which may lead to bias. OTE evaluation allows the individual to integrate all aspects of his condition into a single treatment outcome. It lacks the specific assessment for individual symptoms. A significantly higher rate of patients achieved the first primary end point—namely, improved or extremely improved on the OTE in the previous studies. After trial commencement, the experts suggested that we could use combined end points as the primary outcomes for our study.
3. We deleted the inclusion criterion, “No use of medicine for PDS during two weeks before enrollment”. Because the exclusion criterion, “taking drugs which might affect dyspepsia, such as anti-secretary drugs, antacids, prokinetics, non-steroidal anti-inflammatory drugs and antidepressant drugs before 1 month participating in the trial” overlaps this inclusion.
4. Huguosi Hospital of Chinese Medicine and Beijing Friendship Hospital was added to facilitate recruitment.
5. Advertisements on newspaper and a clinical patient database were added into multimodal strategies.
