# Supplementary material for: Efficacy of acupuncture versus sham acupuncture for postprandial distress syndrome: study protocol for a randomized controlled trial
Source: Trials. 2019 Jan 18;20:65. doi: 10.1186/s13063-018-3051-3 (PMC6339308; doi:10.1186/s13063-018-3051-3)
Supplement: Supplementary file 3 — Members of the Data and Safety Monitoring Board. (DOC 17 kb) [file 13063_2018_3051_MOESM3_ESM.doc]

Additional file 2 Composition of Data and Safety Monitoring Board

| **Name** | **Role on DSMB** | **Affiliation** |
| --- | --- | --- |
| Jian-De Chen, MD | Chair of DSMB voting member | Johns Hopkins Center of Neurogastroenterology |
| Marc Fisher, MD | voting member | Harvard Medical School |
| Wei-Liang Weng, MD | voting member | China Academy of Traditional Chinese Medicine |
| Su-Lun Sun, MD | voting member | China Academy of Traditional Chinese Medicine |
| Jia Liu, MD | voting member | China Academy of Traditional Chinese Medicinev |
